# A novel selenoalkenyl-isoxazole based donor–acceptor nonlinear optical material†
†Electronic supplementary information (ESI) available: Experimental procedure, full analytical and spectral characterization data, and computational parameters. CCDC reference number 1504135. For ESI and crystallographic data in CIF or other electronic format see DOI: 10.1039/c7ce01925d


**DOI:** 10.1039/c7ce01925d

**Published:** 2017-12-05

**Authors:** Brigitte Holzer, Berthold Stöger, Paul Kautny, Georg Reider, Christian Hametner, Johannes Fröhlich, Daniel Lumpi

**Affiliations:** a Institute of Applied Synthetic Chemistry , TU Wien , Getreidemarkt 9/163 , A-1060 Vienna , Austria . Email: brigitte.holzer@tuwien.ac.at ; Email: daniel.lumpi@tuwien.ac.at; b X-Ray Centre , TU Wien , Getreidemarkt 9 , A-1060 Vienna , Austria; c Photonics Institute , TU Wien , Gußhausstraße 27-29 , A-1040 Vienna , Austria

## Abstract

The structure property relationships of untwinned enantiomorphic *Z*-(methylseleno)alkenyl-substituted phenyl-isoxazole and its isostructural triazole congener both crystallizing in the *P*2_1_2_1_2_1_ space group were investigated with respect to UV/vis absorption, thermal behaviour, and second harmonic generation ability. Differential scanning calorimetry revealed a significantly higher melting point of the novel isoxazole compound compared to the triazole derivative and therefore a broader thermal application window. The SHG efficiency of the isoxazole derivative is approximately two thirds the value of the triazole compound and approximately 27 times higher than potassium dihydrogen phosphate. Thus, the developed molecular scaffold represents an interesting novel type of nonlinear optical (NLO) chromophore.

## Abstract 2

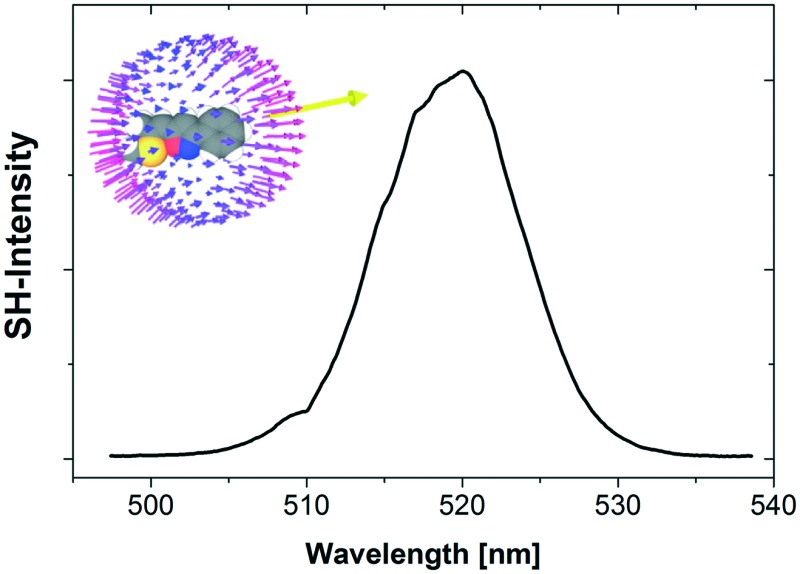
A novel selenium-based donor–acceptor molecule with high second harmonic generation efficiency is presented.

Organic functional materials have become interesting target compounds for optoelectronic applications (such as LEDs, solar cells, and nonlinear optical devices) and constitute inexpensive alternatives to their traditional inorganic semiconductor counterparts.^1^ The fundamental advantage of organic compounds is that the structure property relations of these materials can be tuned toward specific interaction with light in order to process discrete optical signals.^2^ Also, inorganic NLO materials are often expensive in their fabrication due to demanding crystallization processes.^3^ In recent years, a variety of inorganic new second-order NLO crystal materials^4^,^5^ as well as organic push–pull systems asymmetrically linked to π-conjugated spacers exhibiting a high first hyperpolarizability β have been synthesized and probed in electronic and optical applications.^6^–^8^ However, a considerable interest to improve the performance of materials capable of second harmonic generation (SHG) remains. A possible approach towards new compounds may be realized by crystal engineering enabling structure property variations at a molecular level.^9^,^10^

Recently, we have reported on the synthesis and properties of modified ene–yne compounds consisting of *Z*-(alkylthio)alkenyl group as a donor and a triazole moiety as an acceptor (Fig. 1).^11^ Variation of alkyl groups (R′, R′′), isomerization at the double bond, selective sulfur oxidation and introduction of phenyl substituents led to a broad spectrum of nonlinear optical materials.^12^–^14^ The NLO characteristic of this substance class relies on the combination of structural motives of a *Z*-(methylthio)alkenyl group (Fig. 1, ene-substructure) as an electron donor directly connected to an electron-withdrawing heteroaromatic moiety, namely a 1,2,3-triazole, with sufficient acceptor performance. In particular, the modification of the donor–acceptor interaction proved to be advantageous in order to tune the NLO properties. Exchange of sulfur by the more polarizable selenium atom^15^ resulted in highly efficient selenium-substituted 1-phenyl-1,2,3-triazole **2** (Scheme 1), which features a 40 times increased SHG efficiency value compared to potassium dihydrogen phosphate (KDP).^13^ The introduction of electron donating substituents on the phenyl ring (Fig. 1; R=OMe) yielded an NLO active material with approximately 80 times the efficiency of KDP.^14^

**Fig. 1:**
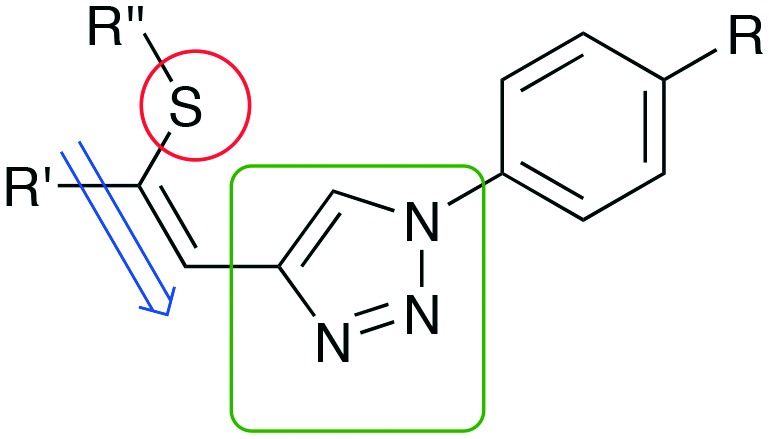
Structural motives for NLO chromophores. Possible structural donor/acceptor modifications include variation of the sulfur donor (red) and the 1,2,3-triazole motive (green).

**Scheme 1:**
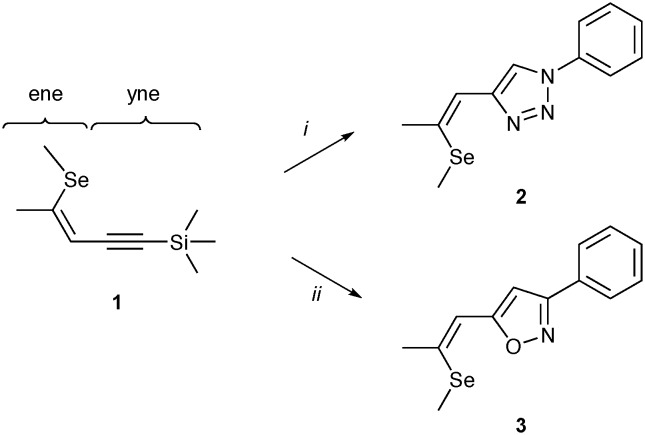
Synthetic approach toward **2** and **3**; i: azidobenzene, CuSO_4_·5H_2_O, Na ascorbate, KF, *t*-BuOH/H_2_O (1 : 1, 0.4 M), MW 150 °C; ii: *N*-hydroxybenzimidoyl chloride, CuSO_4_·5H_2_O, Na ascorbate, KF, *t*-BuOH/H_2_O (1 : 1, 0.4 M), 50 °C.

So far, however, the influence of the electron withdrawing triazole subunit on the NLO properties of the materials has not been investigated. The electron-deficient isoxazole fragment has previously been applied as conjugative π-linker in donor–π–acceptor organic sensitizers.^16^ It represents a structurally related five-membered heterocycle and is an adequate acceptor. This contribution focuses on the structural variation of the triazole-based acceptor subunit in **2** toward material **3** bearing an isostructural isoxazole core as electron accepting moiety (Scheme 1). The presented materials are examined in detail regarding their crystallization behaviour as well as their molecular and NLO properties.

In analogy to the preparation of **2** (ref. 11) the synthesis of compound **3** is based on a modification of the ene–yne compound **1**. A copper(i)-catalyzed nitrile oxide–alkyne cycloaddition allows for a direct functionalization of the yne-substructure (Scheme 1). The cleavage of the trimethylsilyl group in **1** is induced by potassium fluoride giving rise to a terminal alkyne, which subsequently reacts with *in situ* generated phenylnitrile oxide yielding 3,5-disubstituted isoxazole **3** (Scheme 1).

Single crystals of isoxazole **3** were obtained by recrystallization from ethanol (Fig. 2, top).‡
‡Crystal data and refinement details for compound **3**: C_13_H_13_NOSe, *M*_r_ = 278.2, orthorhombic, *P*2_1_2_1_2_1_, *a* = 5.7537(6) Å, *b* = 9.6831(11) Å, *c* = 20.806(2) Å, *V* = 1159.2(2) Å^3^, *Z* = 4, *μ* = 3.216 mm^–1^, *T* = 100 K, 50 130 measured, 5102 independent and 4653 observed [*I* > 3*σ*(*I*)] reflections, 146 parameters, structure solved by charge flipping, refinement against *F*, proton positions fully refined, w*R*_2_ (all data) = 0.0306, *R*_1_ [*I* > 3*σ*(*I*)] = 0.030. The isoxazole **3** and the previously reported triazole **2** (ref. 13) are isostructural and crystallize in the *P*2_1_2_1_2_1_ Sohncke space group, despite being flexible achiral molecules. Hence, single crystals of **3** feature SHG potential as observed for the triazole derivative **2**. The absolute structure and consequently configuration was determined by anomalous dispersion (Flack parameter 0.018(7)). The crystals are generally not twinned, *i.e.* they are composed of only one enantiomorph. The unit cell contains four crystallographically equivalent molecules located on a general position (Fig. 2, bottom). The overall arrangement of the molecule can be described as a herringbone pattern. Intramolecular interactions and torsion angles are summarized in Table 1. An overlay of molecules **2** and **3** reveals that their structures are virtually equivalent (Fig. 3). They adopt a planar configuration, with the phenyl and isoxazole (triazole) ring being coplanar. Only the seleno–methyl group is located slightly out of the molecular plane [C10-C11-Se1-C13 torsion angle of 173.32(17)° (**2**) and 175.24(23)° (**3**)] while the propenyl groups are coplanar with the phenyl ring [angle of least squares plane of phenyl and propenyl fragments: **2**: 4.9(2)°; **3**: 2.01(17)°]. A strong intramolecular interaction in **3** between the selenium and the oxygen atom can be observed (2.867 Å) comparable to the selenium–nitrogen interaction in the triazole compound **2** (2.935 Å).

**Fig. 2:**
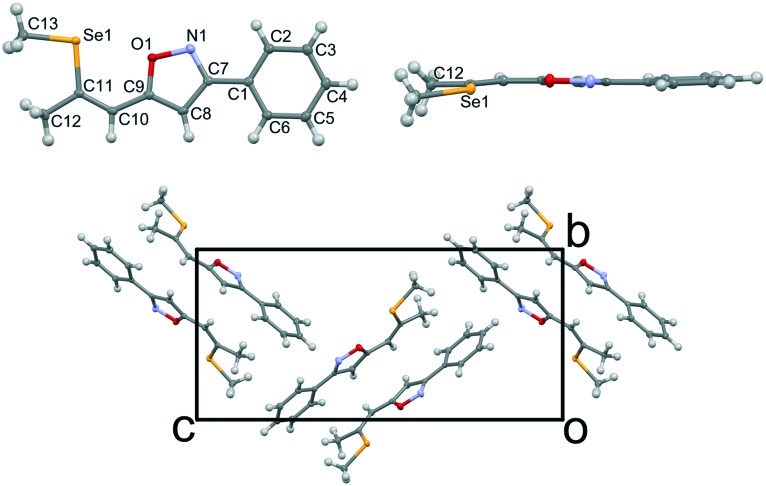
Molecular structure of **3**; viewed normal (top left) and parallel (top right) to the molecular plane. Crystal structure of **3** (bottom). C, N, O and Se atoms are represented by grey, blue, red and orange ellipsoids drawn at the 50% probability levels. H atoms are represented by white spheres of arbitrary radius.

**Table 1:** Intramolecular interactions and torsion angles of **2** and **3**

	Se1···O1/N [Å]	C12-C10-Se1-C13 [°]	Torsion angle benzene-heterocycle [°]
**2**	2.935	173.32(17)	6.83
**3**	2.867	175.24(23)	4.42

**Fig. 3:**
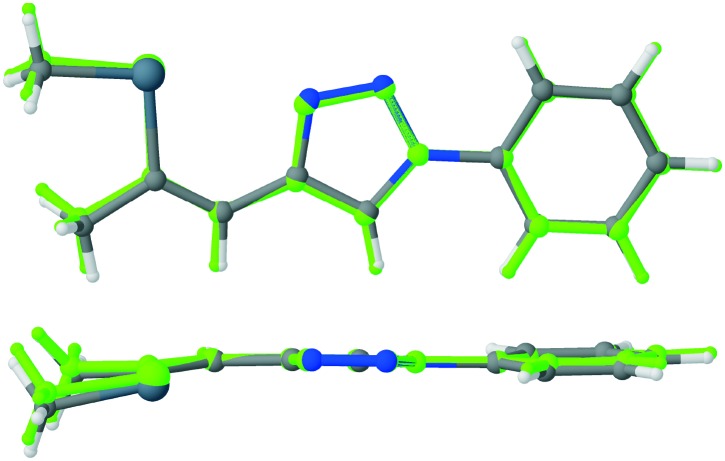
Overlay of the molecular structures of **2** and **3**.

To investigate the photophysical properties of the materials UV/vis absorption spectra of **2** and **3** in tetrahydrofuran were recorded (Fig. 4, top). The absorption onset of **3** is located at 344 nm corresponding to a bandgap of 3.60 eV, whereas **2** exhibits an absorption onset at 332 nm (3.73 eV). Thus, the substitution of the triazole acceptor unit by isoxazole resulted in slightly redshifted absorption onset and decreased optical bandgap. The lower bandgap of the novel NLO chromophore **3** results from a favourable charge transfer induced by intramolecular donor–acceptor interaction.^8^ Due to the absence of any other structural modifications this feature can be directly attributed to the presence of the isoxazole acceptor. Hence, this particular behaviour indicates increased acceptor strength of the isoxazole moiety compared to the triazole. Notably, a high degree of intramolecular charge transfer benefits the macroscopic NLO effect, owing to the high polarizability of the electrons of donor–acceptor materials within the conjugated molecular π-system.^8^,^17^,^18^ Considering the isostructural crystallization behaviour of **2** and **3** a higher NLO activity can thus be expected for **3** compared to **2**.

**Fig. 4:**
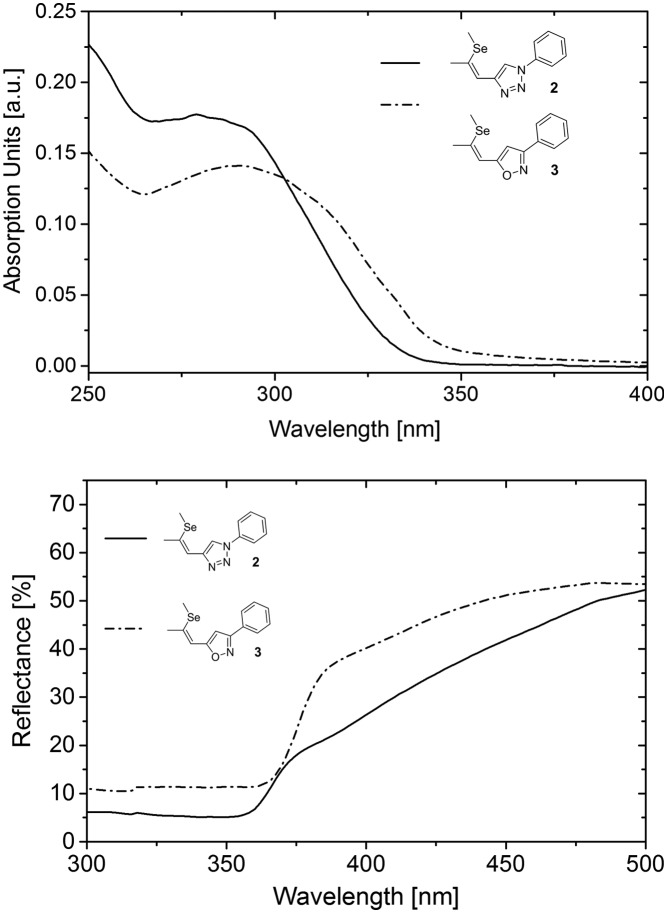
Absorption in solution (top) and optical reflectance spectra of single crystalline probes (bottom) of **2** and **3**.

To investigate the applicability of **3** for SHG generation the optical properties of powdered single crystals were studied. Compared to solution measurements, both **2** and **3** exhibited slightly redshifted absorption onsets. The crystals of **3** feature optical transparency, exhibiting a transmission window above the absorption onset at 398 nm in the visible region (Fig. 4). The absorption spectrum of the novel NLO material **3** in the solid state reveals a redshift of the absorption onset of about 20 nm compared to that of **2**, which may indicate better acceptor properties of the isoxazole compared to the triazole moiety. The thermal properties of **2** and **3** were examined to explore the practical applicability of the materials for SHG. Both materials feature high thermal stability with decomposition temperatures (corresponding to 5% mass loss) higher than 354 °C (Fig. 5, top). DSC analysis (Fig. 5, top, inset) revealed a higher melting point of the isoxazole derivative **3** (104 °C) compared to triazole based **2** (84 °C). Therefore, the newly developed **3** exhibits an improved thermal operation window.

**Fig. 5:**
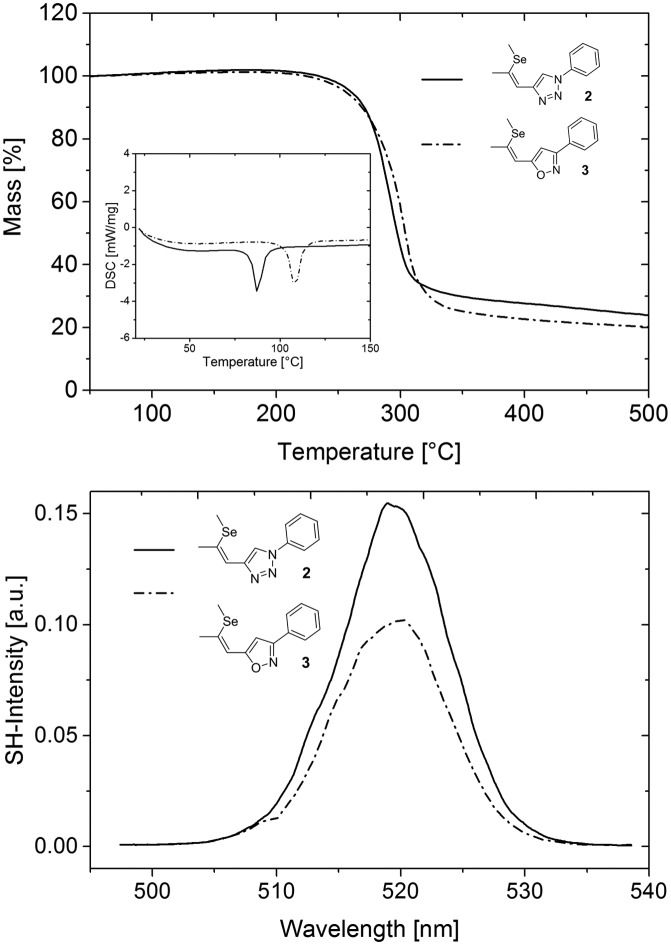
TGA and DSC measurements of **2** and **3** (top). Optical second harmonic spectrum from sub-micron powder samples of **2** (full) and **3** (dash-dot) (bottom); the bandwidth of the signal results from the characteristic output spectrum of the femtosecond laser used for SHG; the irregularities of the spectral shape are due to laser power fluctuations during the measurement of the SH spectrum; the error of the SH peak value resulting from these fluctuations is estimated to be ±10%. Note that because of the quadratic relation *I*^(2*ω*)^*α*(*I*^(*ω*)^*χ*^(2)^)^2^ between the SH signal and nonlinear polarizability *χ*^(2)^, the measured SH ratio of ≈15/10 = 1.5 corresponds to a ratio of the nonlinear polarizabilities of the two substances of ≈√1.5 = 1.25; for the same reason, the estimated error of the polarizability values is about 5%.

Ultimately, the SHG efficiency of **3** was probed, utilizing the powder technique developed by Kurtz and Perry^19^ (for details of the setup see ESI†). The powder was produced by milling the substance with a mortar, and the grain size was established with a microscope to be on the order of the wavelength of the fundamental laser (1034 nm), which is well below the typical nonlinear coherence lengths of transparent media in this wavelength range. Since the present study was restricted to this grain size, no information about possible phase matching is available. For quantification of the SHG yields (*Z*)-4-(2-(methylthio)-1-propenyl)-1-phenyl-1,2,3-triazole^11^ was employed as reference material; the SHG yield of this substance was established to be twice the SHG yield of KDP in an earlier study.^11^ Powdered samples of **3** exhibited intense SHG with an efficiency of approximately 13.5 times the value of the reference material. Accordingly, the observed SH efficiency corresponds to 27 times the value of KDP. Moreover, the SHG yield of **3** is comparable to that of highly efficient isostructural triazole **2** (Fig. 5, bottom). Therefore, the strategy to introduce isoxazole as novel acceptor unit to the parent structure of **2** yielded a novel molecular moiety for the development of SHG materials. Despite the increased donor–acceptor interaction in **3** the SHG efficiency of **3** is lower compared to **2**. Recently, we demonstrated that the analysis of the hyperpolarizabilities of individual molecules and their actual orientation in the single crystal is a useful tool to explain macroscopic SHG efficiency of powdered crystals of organic chromophores.^20^ Consequently, the static first-order hyperpolarizability tensors *β*_*ijk*_ of both molecules were calculated using the Gaussian 09 package. The molecular geometry determined by X-ray diffraction was employed in these calculations (Fig. 6).

**Fig. 6:**
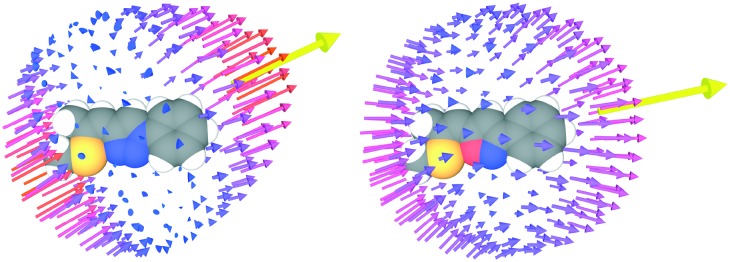
Representation of the first-order hyperpolarizabilities *β*_*ijk*_ of 2 (left) and 3 (right) according to Tuer *et al.*^21^ The quadric response is represented by arrows with the origins located on a sphere around the molecule, whereby the points on the sphere indicate the direction of the applied field. A yellow arrow represents the total first-order hyperpolarizability *β*tot*i*. Its length is scaled by a factor 10 with respect to the other arrows and its origin is placed on the same sphere.

The hyperpolarizability of the crystal was estimated by transforming the atomic coordinates of the single-crystal model into a Cartesian coordinate system. The matrices of direction cosines *a*_*ij*_^22^ transforming the coordinates *x*_*j*_ of the DFT calculation into orthonormal crystal coordinates *x*_*i*_ according to
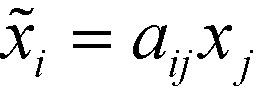
(note: here we use the Einstein summation convention^22^) were determined by minimizing the least-square distances of the corresponding atoms. The *β*_*ijk*_ tensors were then transformed into crystal coordinates by
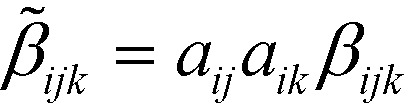
Since both crystals crystallize in the 222 point group, only the six *β̃*_123_ (and permutations of the indices) elements remain. The magnitude of the *β̃*_123_ element of **3** is less than that of **2** (*β̃*_123_ = –1.033 *vs.* 122.7 au) by two orders of magnitude. Thus, these calculations are in qualitative agreement with the SHG measurements, even though the calculated difference is much more pronounced.

A more refined analysis of the hyperpolarizability is necessary to explain the lower magnitude *β̃*_123_ of **3** crystals despite stronger donor–acceptor interactions and identical molecule orientations. The total hyperpolarizability of a molecule can be defined as the vector*β*tot*i* = *β*_*ijj*_

The direction of *β*tot*i* is the average direction of the quadric response. The magnitude |*β*tot*i*| is a measure of the total quadric response, under the precondition that the response is unidirectional [as is the case for **2**, less so for **3** (Fig. 6)]. Indeed, the magnitude |*β*tot*i*| follows the trend expected from the photophysical characterization with 835.1 (**3**) *vs.* 767.6 (**2**) atomic units (au). But whereas *β*tot*i* is close to parallel to the main molecular axis (leading from benzene to the heterocycle) in **2**, it is distinctly inclined in the case of **3** (Fig. 6). For simplicity, we assume at first that the quadric response of the molecules is only observed for field components parallel to *β*tot*i* and the response is likewise parallel to *β*tot*i*. If this direction is defined as [100], the hyperpolarizability tensor is 0 for all elements except *β*_111_. If *a*_*ij*_ transforms this orientation of the molecule into the crystal orientation, *β̃*_123_ = *a*_11_*a*_21_*a*_31_*β*_111_, *i.e. β̃*_123_ is maximized if the *β*tot*i* of the molecules is parallel to a space diagonal.

The *β*tot*i* of both molecules are parallel to the unit vectors (0.14, 0.71, 0.68) (**3**) and (0.37, 0.72, 0.59) (**2**) corresponding to *a*_11_*a*_21_*a*_31_ = 0.07 and 0.16, respectively, compared to a maximum value of *a*_11_*a*_21_*a*_31_ = 
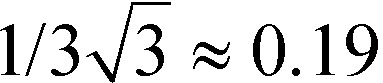
.

Thus, under the assumptions above, 36% and 83% of the maximum possible hyperpolarizability in the 222 crystal system are realized. *β*tot*i* of the molecule is significantly more aligned with the [100] axis in **3**, thus hampering the SHG efficiency.

While these considerations qualitatively confirm the lower calculated ∣*β̃*_123_∣ of the crystal **3**, they cannot explain the two-orders of magnitude in difference. Thus, one has to consider the non-unidirectionality of the hyperpolarizability. If the tensors of both molecules are transformed such that *β*tot*i* is parallel to [100] direction, they read in the commonly used piezoelectric 3 × 6 *β*_*ij*_ matrix form *β*_*i*1_ = *β*_*i*11_, …, *β*_*i*6_ = 2*β*_*i*12_ as
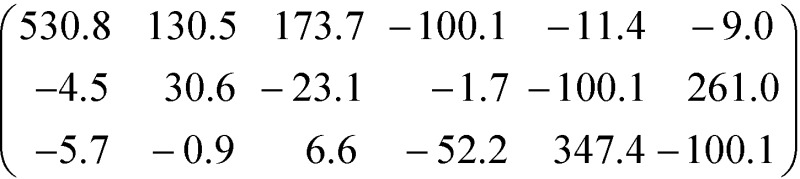
and
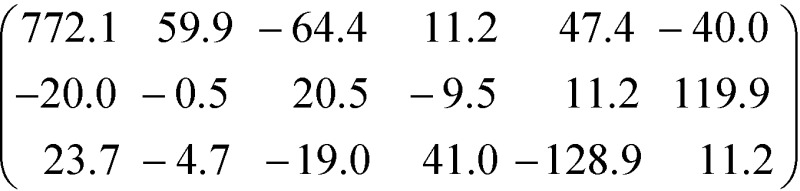


Surprisingly, for **3** the *β*_111_ element is distinctly smaller, despite a larger |*β*tot*i*| owing to distinct hyperpolarization which is not parallel to the main polarization axis. On transformation into crystal coordinates, the corresponding non-*β*_111_ tensor elements significantly reduce the *β̃*_123_ element.

In summary, the lower SHG efficiency of **3** is attributed to two orthogonal phenomena, *viz.* an alignment of the total hyperpolarizability with the [100] axis, and a significant divergence of the quadric response.

## Conclusions

In conclusion, we report on a novel selenoalkenyl-isoxazole based donor–acceptor material, which exhibits enantiomorphic crystallization and second-order nonlinear optical properties with an estimated efficiency of second harmonic generation approximately 27 times the value of potassium dihydrogen phosphate. While the second harmonic generation efficiency of the new material is slightly lower compared to an analogous triazole-based derivative, its thermal operation window is improved. Therefore, the presented π-electron conjugated combination of phenylisoxazole and *Z*-(methylseleno)alkenyl moieties represents an efficient method for the design of functional donor–acceptor-based organic materials and a novel molecular structure for the development of non-linear optical materials. Furthermore, our in depth analysis of the hyperpolarizability of the materials under investigation revealed, that the evaluation of exclusively molecular properties is insufficient to estimate the observable second harmonic generation. Even in the case of isostructural derivatives, the exact shape of the first-order hyperpolarizability tensor has to be carefully considered in order to obtain a meaningful estimate for the macroscopic non-linear effect.

## Conflicts of interest

There are no conflicts to declare.

## Supplementary Material

Supplementary information

Crystal structure data
