# Supplementary material for: A novel selenoalkenyl-isoxazole based donor–acceptor nonlinear optical material
Source: CrystEngComm. 2017 Dec 5;20(1):12–6. doi: 10.1039/c7ce01925d (PMC5885368; doi:10.1039/c7ce01925d)
Supplement: Supplementary file 1 [file CE-020-C7CE01925D-s001.pdf]

Electronic Supplementary Material (ESI) for CrystEngComm.
This journal is © The Royal Society of Chemistry 2017

Electronic Supplementary Information

A novel selenoalkenyl-isoxazole based donor-acceptor nonlinear optical material

Brigitte Holzer,*[a] Berthold Stöger,[b] Paul Kautny,[a] Georg Reider,[c] Christian Hametner,[a] Johannes Fröhlich,[a] and Daniel Lumpi*[a]

[a] Institute of Applied Synthetic Chemistry, TU Wien, Getreidemarkt 9/163, A-1060 Vienna, Austria.

[b] Institute of Chemical Technologies and Analytics, TU Wien, Getreidemarkt 9/164, A-1060 Vienna, Austria

[c] Photonics Institute, TU Wien, Gußhausstraße 27-29, A-1040 Vienna, Austria

*brigitte.holzer@tuwien.ac.at

*daniel.lumpi@tuwien.ac.at

Table of contents:

1. Synthesis
2. NMR Data
3. Crystallographic Part
4. Powder SHG Studies
5. Theoretical Calculations
6. References

The following reaction was performed in oven-dried glassware. Reagents were purchased from commercial sources and used without prior purification. Analytical TLC was performed on Merck silica gel 60 F254 plates. Chromatographic separations at preparative scale were carried out on silica gel (Merck silica gel 60, 40 – 63 µm). UV/Vis absorption spectra were recorded in tetrahydrofuran solutions (5 µM) or for powder samples using Suprasil glass slides as sample holder with a Perkin Elmer Lambda 750 spectrometer. The thermal behaviour of substances **2** and **3** was studied with differential scanning calorimetry (DSC) and thermogravimetric analysis (TGA) using a Netzsch simultaneous thermal analyzer (STA 449 F1 Jupiter) under N_2 flow (40 mL/min) with a heating rate of 10 K/min. The STA 449 Type-K thermocouples were calibrated using indium, tin, bismuth and zinc metals. The syntheses of trimethyl[(3*Z*)-4-(methylseleno)-3-penten-1-yn-1-yl]silane **1**[1] and *N*-hydroxybenzimidoyl chloride **4**[2] were performed according to literature.

Nuclear magnetic resonance (NMR) spectra were recorded on a Bruker Avance III HD 600 MHz spectrometer equipped with a cryo probe Prodigy™ at 600.2 MHz (^{1}H), 150.9 MHz (^{13}C) and 114.5 MHz (^{77}Se). The ^{1}H and ^{13}C chemical shifts are reported in δ units, parts per million (ppm) downfield from tetramethylsilane using residual solvent signals for calibration. ^{77}Se chemical shifts are given in ppm relative to dimethylselenide, using selenophene (δ = 605 ppm)[3] as an external secondary standard. Coupling constants are reported in Hertz; multiplicity of signals is indicated by using following abbreviations: s=singlet, d=doublet, t=triplet, q=quartet, quin=quintet. The multiplicity of ^{13}C signals was obtained by measuring JMOD spectra. The high-resolution mass spectrum (HRMS) was acquired using a Thermo Scientific LTQ Orbitrap XL hybrid FTMS (Fourier Transform Mass Spectrometer) equipped with Thermo Fisher Exactive Plus Orbitrap (LC-APCI+) and a Shimadzu IT-TOF Mass Spectrometer.

1. Synthesis

5-[(1*Z*)-2-(Methylseleno)-1-propen-1-yl]-3-phenylisoxazole (**3**). To a t-BuOH/H_2O (1:1, 0.4 M) suspension of **1** (231 mg, 1 mmol), *N*-hydroxybenzimidoyl chloride **4** (171 mg, 1.1 mmol), $CuSO_4.5H_2O$ (5 mg, 0.02 mmol), sodium ascorbate (20 mg, 0.1 mmol), and potassium fluoride (71 mg, 1.2 mmol) were added. Subsequently, the reaction mixture was heated at 50 °C for 60 min, diluted with water and extracted with Et_2O. The combined organic layers were washed with brine and dried over anhydrous Na_2SO_4. Filtration and evaporation of the solvent followed by column chromatography (light petroleum / ethyl acetate 7:1) yielded **3** (141 mg, 51%) as a colourless solid. Single crystals were obtained by crystallization from a saturated solution in ethanol. R_f = 0.50 (light petroleum : ethyl acetate 5 : 1). ^{1}H NMR (600 MHz, CD_2Cl_2): δ = 7.83 – 7.81 (m, 2 H), 7.49 - 7.44 (m, 3 H), 6.72 (q, J = 1.3 Hz, 1 H), 6.70 (s, 1 H), 2.37 (d, J = 1.3 Hz, 3 H), 2.32 (s, 3 H) ppm. ^{13}C NMR (150 MHz, CD_2Cl_2): δ = 168.5 (s), 162.3 (s), 140.8 (s), 129.9 (d), 129.4 (s), 128.9 (d), 126.8 (d), 112.5 (d), 99.9 (d), 25.7 (q), 5.21 (q) ppm. ^{77}Se NMR (114 MHz, CD_2Cl_2): δ = 246 ppm. HR-ESI-FTMS $[M+H]^+$ m/z calcd. 280.0235 for $C_{13}H_{14}NOSe^+$, found 280.0231.

2. NMR Data

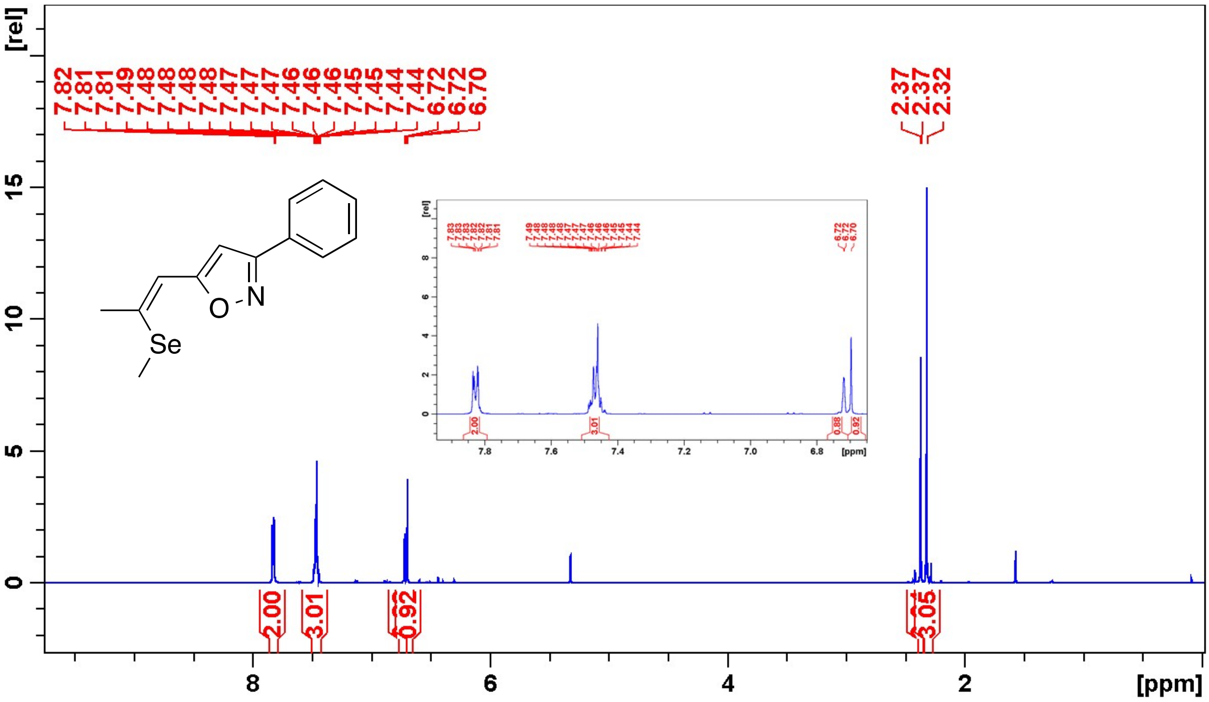

Figure S1. ^{1}H NMR (600 MHz, CD_2Cl_2) of compound **3**.

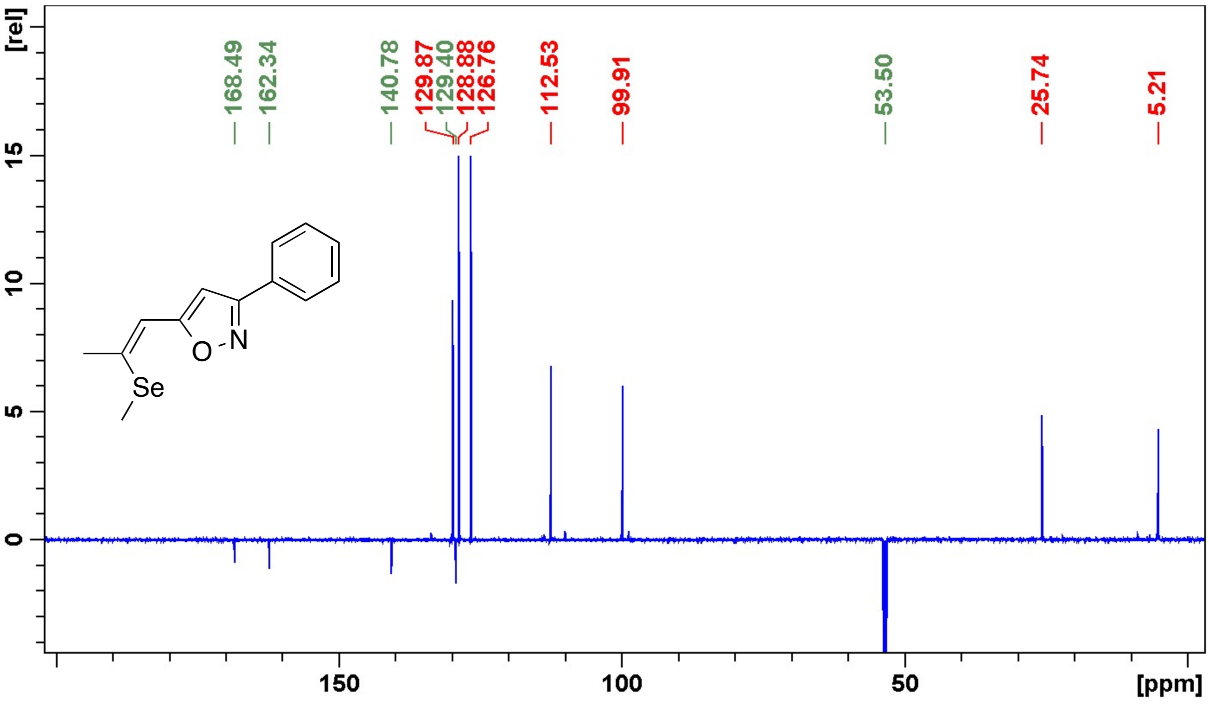

Figure S2. ^{13}C NMR (150 MHz, CD_2Cl_2) of compound **3**.

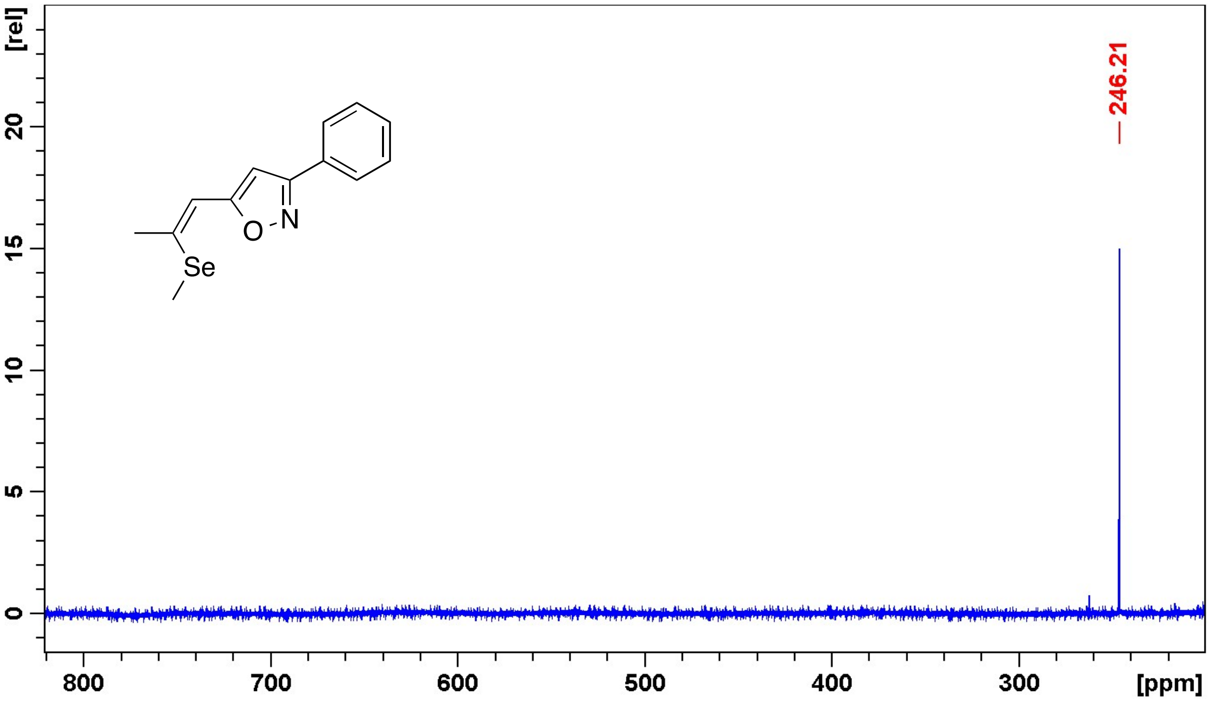

Figure S3. ^{77}Se NMR (114 MHz, CD_2Cl_2) of compound **3**.

3. Crystallographic Data

X-ray diffraction intensities of a suitable crystal of the title compound **3** were collected at 100 K in a dry stream of nitrogen on a Bruker KAPPA APEX II diffractometer system using graphite-monochromatized Mo-Kα radiation (λ = 0.71073 Å) and fine sliced φ- and ω-scans. Frame data were reduced to intensity values with SAINT-Plus[4] and a correction for absorption effects was applied using the multi-scan approach implemented in SADABS[4]. More details of the data collection are summarized in Table 1. The crystal structure was solved by charge-flipping using SUPERFLIP.[5] All non-H atoms were located in the resulting electron density map. The structure was refined with JANA2006.[6] The protons were placed at calculated positions and refined as riding on their parent atoms. All non-H atoms were modelled with anisotropic displacement parameters.

Table 1. Details of single crystal data collection and structure refinement of **3**.

Diffractometer	Bruker SMART APEX II
Radiation; λ / Å	Mo Kα; 0.71073
Temperature / °C	-173
Crystal colour; shape	clear colourless rod
Space group, no.	$P\,2_1\,2_1\,2_1$
Formula units Z	4
a / Å	5.7537(6)
b / Å	9.6831(11)
c / Å	20.806(2)
V / Å^3	1159.2(2)
Sum formula	$C_{13}H_{13}NOSe$
Formula weight	278.2
μ / mm^{-1}	3.216
X-ray density / g·cm^{-3}	1.5942
Range θ_{min}- θ_{max}	1.96 – 35.02
Range h	-9 → 9
k	-15 → 15
l	-33 → 33
Measured reflections	50130
Independent reflections	5102
Obs. Reflections [I>3σ(I)]	4653
R_i	0.034
Absorption correction	SADABS
Number of parameters	146
Diff. elec. dens. max; min [e-· Å^{-3}]	0.80; -0.66
R[F>3σ(F)]	0.0300
wR(F^2 all)	0.0306
GooF	1.52
Flack Parameter	0.018(7)

4. Powder SHG Studies

For a qualitative evaluation of the NLO properties of **3**, the process of frequency doubling or second harmonic generation (SHG) was employed utilizing the powder technique developed by Kurtz and Perry.[7] This method allows the relative measurement of the NLO coefficients since the SH efficiency scales quadratically with the nonlinear coefficient for samples with a particle size that is significantly less than the so-called nonlinear coherence length (which is more than 10 μm). Powdered samples with a grain size of less than 1 μm were prepared by grinding in a mortar. These freshly prepared approximately 200 μm thick samples, sandwiched between microscope slides, were irradiated with the output of an ultrafast Yb:KGW-Laser (light conversion, pulse duration 70 femtoseconds, average power 400 mW, repetition rate 75 MHz, wavelength 1034 nm), moderately focused with a 100 mm focusing lens, impinging onto the sample under an angle of incidence of 50°. The diffusely reflected SH radiation was collected with a NA = 0.1 lens, separated from the fundamental radiation with a color filter, and spectrally analyzed with a 0.25 m grating monochromator and a photomultiplier detector in the range between 450 and 600 nm. The angle between the optical axis of the monochromator and the sample surface normal was 40°, so that no specular reflection from the glass slides was incident on the monochromator input. The resulting SH spectrum was identical to that obtained by inserting a commercial SH crystal into the beam path, displaying the peak at 1034 nm / 2 = 517 nm and an FWHM of 6 nm, which is due to the relatively broad spectrum of the femtosecond laser source. The sample plane was positioned somewhat out of the focal plane (toward the lens) so as to prevent any damage to the sample. After each measurement, the samples were carefully checked for the absence of damage or thermal modification. The SH signal of our sample was compared to that from a similarly prepared powder sample of another organic compound[8] with point group 222, which exhibits a second-order nonlinear coefficient of 0.6 x 10^{-12} m V^{-1}, somewhat higher than potassium dihydrogen phosphate (KDP).

5. Theoretical calculations

DFT calculations were performed using the Gaussian 09 package revision D.01.[9] applying the long range corrected CAM-B3LYP functional[10] in combination with Pople basis sets 6-311++G(d,p)[11,12] in order to include polarized and diffuse functions. Geometry optimizations were performed in the gas phase. First-order hyperpolarizability tensors were visualized using a method adapted from Tuer *et al.*[13] The graphical representations were rendered using the POVRAY software package.[14]

6. References

[1] D. Lumpi, F. Glöcklhofer, B. Holzer, B. Stöger, C. Hametner, G. A. Reider and J. Fröhlich, *Cryst. Growth Des.*, 2014, **14**, 1018.

[2] P. Y. S. Lam, J. J. Adams, C. G. Clark, W. J. Calhoun, J. M. Luettgen, R. M. Knabb and R. R. Wexler, *Bioorg. Med. Chem. Lett.*, 2003, **13**, 1795.

[3] L. Christiaens, J.-L. Piette, L. Laitem, M. Baiwir, J. Denoel and G. Llabres, *Org. Magn. Reson.* 1976, **8**, 354.

[4] Bruker Analytical X-ray Instruments, Inc., Madinson, WI, USA: SAINT and SADABS 2008.

[5] L. Palatinus, G. Chapuis, G. *J. Appl. Cryst.* **2007**, 40, 786.

[6] V. Petříček, M. Dušek, L. Palatinus, **2006**: Jana2006. The crystallographic computing system. Institute of Physics, Praha, Czech Republic.

[7] S. K. Kurtz, *J. Appl. Phys.* **1968**, 39, 3798.

[8] A. A. Kaminskii, L. Bayarjargal, E. Haussühl, N. Müller, S. Haussühl, H. J. Eichler, H. Rhee, K. Ueda, A. Shirakawa, G. A. Reider, *Phys. Status Solidi A*, **2011**, 208, 695–709.

[9] M. J. Frisch, G. W. Trucks, H. B. Schlegel, G. E. Scuseria, M. A. Robb, J. R. Cheeseman, G. Scalmani, V. Barone, B. Mennucci, G. A. Petersson, H. Nakatsuji, M. Caricato, X. Li, H. P. Hratchian, A. F. Izmaylov, J. Bloino, G. Zheng, J. L. Sonnenberg, M. Hada, M. Ehara, K. Toyota, R. Fukuda, J. Hasegawa, M. Ishida, T. Nakajima, Y. Honda, O. Kitao, H. Nakai, T. Vreven, J. A. Montgomery Jr., J. E. Peralta, F. Ogliaro, M. J. Bearpark, J. Heyd, E. N. Brothers, K. N. Kudin, V. N. Staroverov, R. Kobayashi, J. Normand, K. Raghavachari, A. P. Rendell, J. C. Burant, S. S. Iyengar, J. Tomasi, M. Cossi, N. Rega, N. J. Millam, M. Klene, J. E. Knox, J. B. Cross, V. Bakken, C. Adamo, J. Jaramillo, R. Gomperts, R. E. Stratmann, O. Yazyev, A. J. Austin, R. Cammi, C. Pomelli, J. W. Ochterski, R. L. Martin, K. Morokuma, V. G. Zakrzewski, G. A. Voth, P. Salvador, J. J. Dannenberg, S. Dapprich, A. D. Daniels, Ö. Farkas, J. B. Foresman, J. V. Ortiz, J. Cioslowski and D. J. Fox, *Gaussian 09*, Gaussian, Inc., Wallingford, CT, USA, 2009.

[10] T. Yanai, D. P. Tew and N. C. Handy, *Chem. Phys. Lett.*, 2004, **393**, 51.

[11] R. Krishnan, J. S. Binkley, R. Seeger and J. A. Pople, *J. Chem. Phys.*, 1980, **72**, 650.

[12] M. J. Frisch, J. A. Pople and J. S. Binkley, *J. Chem. Phys.*, 1984, **80**, 3265.

[13] A. Tuer, S. Krouglov, R. Cisek, D. Tokarz and V. Barzda, *J. Comput. Chem.*, 2011, **32**, 1128.

[14] Persistence of Vision Raytracer (Version 3.6) Persistence of Vision Pty. Ltd., Williamstown, Victoria, Australia.
